# Expanding Free Open-Access Medical Education

**DOI:** 10.3389/fmed.2021.794667

**Published:** 2021-12-23

**Authors:** Akhil Bansal

**Affiliations:** Faculty of Medicine, University of Sydney, Sydney, NSW, Australia

**Keywords:** medical education, e-learning (web based learning/distance learning), faculty development, continuing medical education (CME), virtual learning and education environment (VLE)

During my medical school cardiology placement, I remember our clinical tutor printing out the cardiovascular examination checklist from *Geeky Medics*^©^ as our learning tool for the term. When I would study later for my pre-clinical exams, *Amboss*^©^ and *UpToDate*^©^ were the first and most useful resources that I looked at, rather than my course lectures. When I would study for finals, the advice from seniors was to get through the core lectures from our university quickly, and then to move onto online question banks. My experience was not unique; I would rather confidently say it characterized the learning and revision approach of most of my peers.

These examples are not to point out the failings of my medical school; it taught and prepared me exceptionally well, and I received a very high quality of medical education and support. Rather, it reflects a broader shift within medical education. Ebbing are the days of learning from the 1-h, in-person, didactic teaching style that is the cornerstone of medical education. Flowing are the days of open-access, online learning resources.

Over the last decade, there has been considerable growth in free open-access medical education (FOAM), which refers to openly accessible and predominantly online materials used to supplement and enhance traditional educational methods. FOAM includes teaching resources such as blogs, podcasts, tweets, online question banks, YouTube videos, and other social media platforms that are easily and freely available.

Over 90% of both pre-clinical (1) and clinical (2) medical students use online FOAM resources on a weekly basis, and it is a core source of core learning and revision material for students. This information crosses institutional and geographical borders, and allows for the global, continuous, and instantaneous spread of clinical knowledge. In doing so, FOAM has the unique ability to promote the equity (3) and quality of medical education, and more broadly promote global health equity. Especially considering the COVID-19 pandemic, the need for clinical knowledge to be easily and quickly disseminated has been highlighted.

As such a predominant and important teaching pedagogy, its current landscape and areas for development need to be considered. This article will first discuss what FOAM exists, justify the importance of faculty development in FOAM and offer a set of recommendations for what faculty development in FOAM should exist.

## The Current Landscape of Free, Open Access Medical Education

FOAM resources are concentrated in their geographical origin and content domain; most resources are created in a small number of high-income countries: Australia, UK, and USA (4). This is despite the fact the users of FOAM resources are from a diverse range of high-, middle- and low-income countries around the world (4), and that most doctors work in middle- and low-income countries. Further, although expanding, there are certainly clinical domains that are over and under-represented. Emergency and critical care medicine (5) and pediatric medicine have both been pioneers in the FOAM medicine, whereas a number of other clinical specialities have a death of resources. Both these factors may potentially result in poor alignment of FOAM resources to the educational needs of students around the world.

In addition, most resources are developed by dedicated individual clinicians independent of an academic institution. A notable excision to this is massive open online courses (MOOCS), which are generally developed by universities and available through several platforms (such as *Coursera*^©^ and *edX*^©^), However, MOOCS for medical students are scarce, and they are overall not as comprehensive or widely used as other FOAM tools.

## Potential Concerns With Foam

As a new learning pedagogy, there are potential barriers and challenges regarding the development, design, and evaluation of FOAM resources. The four main challenges are:

1. A lack of motivation, skills, and experience- The most significant barrier is the perceived difficulty and challenges in making FOAM resources, with clinical educators describing a lack of technical expertise, time, resources and motivation (6).

2. Best practice for instructional design- There is concern regarding best practice for the instructional design and pedagogical frameworks for FOAM resources. For example, although podcasts are frequently used by doctors to review new literature and learn core material, most did not perform active learning whilst listening to the podcast, limiting their retention of information (7). Since the pedagogical design can affect the effectiveness of FOAM (8), this must be carefully considered to ensure they are beneficial.

3. Evaluation of FOAM resources- It may be difficult to verify the legitimacy and accuracy of information of resources produced online (9). Further, there is limited literature on how to discern high quality FOAM material. Although there are some metrics and tools that have been established to score FOAM resources (10), these have not been validated and widely adopted at this time.

4. Sustainability- There are concerns regarding the sustainability of FOAM materials. Their creation is currently being driven by individual clinicians and is hence contingent on their altruism and availability. As a result of this, it is feared that FOAM resources may not be a sustainable and comprehensive teaching pedagogy long-term.

## Faculty Development in Online Learning Resources

The growing use of FOAM resources and some of the potential concerns regarding their use justifies the need for academic institutes to invest and take an active role in developing and evaluating them. Faculties should consider developing FOAM resources to empower their faculty in adopting a new era and dominant pedagogy in medical education. This will not only benefit their own students, who are increasingly turning to such resources for their education, but also help these faculties become more active members in the global community of medical educators.

Faculty development programs in developing FOAM resources are likely to play an instrumental role in overcoming the challenges mentioned above and ensuring the creation of more high-quality resources, as summarized in Table 1.

**Table 1:** The challenges in the implementation of free, open access medical resources, and the role of faculty development programs in addressing them.

**Challenge**	**Role of faculty development programs**
Lack of motivation, skills, and experience	Faculty development programs would offer clinical educators background on the importance of FOAM and the tools and confidence to create resources
Best practice for instructional design	Formal training in creating FOAM resources would offer a pedagogical framework to inform the design of appropriate and useful FOAM resources
Evaluation of FOAM resources	Resources made by medical faculties would go through their institutional checks and verifications
Sustainability	Faculty development programs would train a cohort of clinical educators who can drive the creation of FOAM resources

Several medical faculties have developed faculty programs in developing e-learning resources and FOAM education more broadly. However, of the few programs that do currently exist, they tend to be institution-specific, paid, and in-person, counter-intuitive to the principle of open-access.

In light of this, this article makes the following suggestions:

1. Free, open access medical education (FOAM) is increasingly becoming an important teaching pedagogy, and medical faculties should therefore consider creating FOAM resources.
2. To achieve this, implementing faculty development programs in developing FOAM resources are necessary.
3. Faculty development programs should themselves be free, open access, and available online.

## Author Contributions

The author confirms being the sole contributor of this work and has approved it for publication.

## Conflict of Interest

The author declares that the research was conducted in the absence of any commercial or financial relationships that could be construed as a potential conflict of interest.

## Publisher's Note

All claims expressed in this article are solely those of the authors and do not necessarily represent those of their affiliated organizations, or those of the publisher, the editors and the reviewers. Any product that may be evaluated in this article, or claim that may be made by its manufacturer, is not guaranteed or endorsed by the publisher.
